# Bullying as a Risk for Poor Sleep Quality among High School Students in China

**DOI:** 10.1371/journal.pone.0121602

**Published:** 2015-03-26

**Authors:** Ying Zhou, Lan Guo, Ci-yong Lu, Jian-xiong Deng, Yuan He, Jing-hui Huang, Guo-liang Huang, Xue-qing Deng, Xue Gao

**Affiliations:** 1 Department of Medical Statistics and Epidemiology, School of Public Health, Sun Yat-sen University, Guangzhou, China; 2 Center for ADR monitoring of Guangdong, Guangzhou, China; 3 Center for Disease Control and Prevention of Haizhu District, Guangzhou, China; Weill Cornell Medical College in Qatar, QATAR

## Abstract

**Objective:**

To determine whether involvement in bullying as a bully, victim, or bully-victim was associated with a higher risk of poor sleep quality among high school students in China.

**Methods:**

A cross-sectional study was conducted. A total of 23,877 high school students were surveyed in six cities in Guangdong Province. All students were asked to complete the adolescent health status questionnaire, which included the Chinese version of the Pittsburgh Sleep Quality Index (PSQI) and bullying involvement. Descriptive statistics were used to evaluate sleep quality and the prevalence of school bullying. Multi-level logistic regression analyses were conducted to examine the association between being victimized and bullying others with sleep quality.

**Results:**

Among the 23,877 students, 6,127 (25.66%) reported having poor sleep quality, and 10.89% reported being involved in bullying behaviors. Of the respondents, 1,410 (5.91%) were pure victims of bullying, 401 (1.68%) were bullies and 784 (3.28%) were bully-victims. Frequently being involved in bullying behaviors (being bullied or bullying others) was related to increased risks of poor sleep quality compared with adolescents who were not involved in bullying behaviors. After adjusting for age, sex, and other confounding factors, the students who were being bullied (OR=2.05, 95%CI=1.81-2.32), bullied others (OR=2.30, 95%CI=1.85-2.86) or both (OR=2.58, 95%CI=2.20-3.03) were at a higher risk for poor sleep quality.

**Conclusions:**

Poor sleep quality among high school students is highly prevalent, and school bullying is prevalent among adolescents in China. The present results suggested that being involved in school bullying might be a risk factor for poor sleep quality among adolescents.

## Introduction

High school students are special populations that endure a period of great challenges, risks and social developmental transitions. Numerous studies have confirmed the importance of sleep in behavioral regulation during the development of adolescents [1, 2]. Sleep quality is an important clinical construct and essential part of quality of life. Numerous studies have shown that poor sleep quality can place adolescents under the negative influences of impaired cognitive function, poor academic performance, depression, alcohol consumption, and suicidal behavior [3, 4]. Currently,poor sleep quality may be highly prevalent among high school students, and studies have shown that the number of adolescents involved in poor sleep quality worldwide varies between 18.7% and 25%[5–7]. However, the causes of poor sleep are complex and certainly multifactorial. Studies have reported that age, female sex, low socioeconomic status, living alone, and some environmental and occupational factors, as well as poor mental and psychological health, may be risk factors for having a sleep disorder [8–11].

Research has been undertaken that targets poor sleep quality among school students. The association between school behavior and sleep has been considered to be worth studying because school behavior may be strongly related to sleep and sleep problems. School bullying is a form of aggressive behavior experienced in schools that is defined as repeated exposure to negative actions by one or more schoolmates over time [12]. There are four profiles associated with bullying: pure bullies (students who bully and are never victims), pure victims (students who are victims of bullying and never attack others), bully-victims (students who are both victims of bullying and who bully others) and neutral (students never involved in bullying) [13]. Studies have shown that the number of students involved in this type of peer relationship in different countries varies between 6.3% and 45.2%, suggesting that bullying is prevalent among adolescents in schools [14, 15]. Numerous studies have shown that bullying affects the health of adolescents in the same way worldwide and appears to be linked to sleep quality [16, 17]. Williams *et al* found that children who were frequently bullied at school were more likely to get enuresis (OR = 1.7, 95%CI = 1.3–2.4) and have difficulty sleeping (OR = 3.6, 95%CI = 2.5–5.2) [18]. Previous studies have shown that the victims of bullying are more likely to have sleep difficulties among both women (OR = 1.47, 95% CI = 1.26–1.72) and men (OR = 1.58, 95%CI = 1.06–2.36) [19]; those victims also have a lower sleep quality [20] and more often use sleep medication [21] compared with non-bullied respondents. Another study [19, 22] showed that past exposure to bullying increased the risk of sleep disturbances among women (OR = 3.83, 95%CI = 3.12–4.70) and men (OR = 4.40, 95%CI = 3.35–5.78) in the workplace, after adjusting for covariates. These studies, however, were conducted on relatively small or selective samples, examined nonstandard measures for the assessment of sleep quality, or did not take adequate account of potential confounding factors such as socio-demographic factors. Furthermore, little is known about the sleep quality that may be associated with each bullying profile (pure victim, pure bully, bully-victim and neutral) among high school students.

In addition, most of the previous studies that measured sleep quality in China only used simple unstructured questions, which may be insufficient to deliver valid assessments of sleep quality. The Pittsburgh Sleep Quality Index (PSQI) was designed to evaluate sleep quality through 19 items on multiple dimensions of sleep over a 1-month period and has been proven to have good reliability and test-retest reliability [23]. It assesses seven aspects of sleep, including subjective sleep quality, sleep latency, sleep duration, habitual sleep efficiency, sleep disturbances, use of sleeping medication, and daytime dysfunction [24]. Examining several aspects of sleep simultaneously in the same sample could provide a more precise and exhaustive description of the different bullying profiles and the challenges faced by the students involved in bullying.

Currently, we do not know whether bullying behavior in high school is an independent risk factor for poor sleep quality among high school students. The present study was designed to fill this gap in knowledge. The present study explored the different aspects of sleep (e.g., duration, latency and habitual sleep efficiency) of the four bullying profiles (pure victim, pure bully, bully/victim and neutral) using the validated PSQI by determining whether students who experience frequent bullying behaviors in high school (as bullies, victims or both) are at risk for poor sleep quality.

Furthermore, the sample of previous studies about sleep quality in other countries ranged from 124 to 3906 [25]. Concerning the sleep quality status quo among adolescents and the relatively small sample of previous studies, the present study conduct a large-scale, randomly selected sample investigated the prevalence of school bullying and sleep quality among Chinese high school students.

## Methods

### Participants

A cross-sectional study was conducted to investigate the prevalence of school bullying and sleep quality among high school students. The participants were high school students recruited from six cities in the Guangdong Province through a three-stage stratified group sampling method. For the first stage, the cities were divided into three strata according to their economic level, and two cities were selected in each stratum. For the second stage, 12 schools (6 junior high schools, 4 senior high schools and 2 vocational high schools) were randomly selected from all the schools in each city. For the third stage, 2 classes were randomly selected from each grade in the selected schools. All students (a total of 25,655) in the selected classes were asked to participate in this study and provided informed consent voluntarily. A total of 23,877 valid questionnaires were collected, resulting in a valid rate of 93.36%.

### Data Collection

To protect the privacy of the students, anonymous questionnaires were administered by trained interviewers in the absence of the teachers (to avoid any potential information bias). The students were required to fill out the questionnaires during class time.

### Ethical Statement

This study received approval from the Sun Yat-Sen University, School of Public Health Institutional Review Board. All participants were fully informed of the purpose of the study and were invited to participate voluntarily. Written consents were obtained from each participant and their parents.

### Measures

#### Demographic characteristics

The demographic questionnaire elicited information on age, gender, grade, economic status, academic pressure, relationships with families, classmates and teachers, and smoking. Family economic status was measured by asking the student’s perception of their family’s current status (rated from good to bad). Academic pressure was captured by a single item based on the student’s self-rating from low to high. Relationships with families, classmates and teachers were assessed based on the student’s self-rating from good to poor. Smoking was assessed by asking, “Did you smoke at least one cigarette per week?”

#### Sleep quality

The students’ sleep quality was measured by the Chinese version of PSQI, which consists of 19 items generating 7 components: subjective sleep quality; sleep latency; sleep duration; habitual sleep efficiency; sleep disturbances; use of sleep medication; and daytime dysfunction. The CPSQI had been proven to have a good overall reliability and test-retest reliability. The CPSQI has a cutoff value of 7, with higher global scores indicating poorer sleep quality [24].

#### Bullying behaviors

There are four profiles associated with bullying: pure bullies (students who bully and are never victims), pure victims (students who are victims of bullying and never attack others), bully-victims (students who are both victims of bullying and who bully others) and neutral (students never involved in bullying). The questions about bullying and victimization consisted of 12 parts, with the answers given on a 3-point scale as follows: 1-never, 2-sometimes (one or two times) or 3-often (more than three times). Students reporting at least one bullying behavior with a frequency of ‘‘often” in the past 30 days were classified as bullies [26]. Victims were those who reported at least one victimization experience in the past 30 days with a frequency of ‘‘often.” Bully-victims met the criteria for being both a bully and a victim. All other students were labeled as neutral [27].

#### Bullying questionnaire

The questions about bullying and victimization consisted of 12 parts, with the answers given on a 3-point scale as follows: 1-never, 2-sometimes (one or two times) or 3-often (more than three times).

Bullying and victimization were assessed with parallel questions: “During the last 30 days have you ever been (a1) ‘‘hit, kicked, pushed, shoved around, or locked another student indoors?”; (b1) ‘‘made fun of or insulted?”; (c1) ‘‘excluded intentionally or prevented from participating?”; (d1) ‘‘made fun of with sexual jokes, comments or gestures?”; (e1) ‘‘blackmailed for money?” or (f1) ‘‘bullied in some other way?”. Question for bullying were as follows: Have you ever (a2) ‘‘hit, kicked, pushed, shoved around, or locked another student indoors?” (b2) ‘‘made fun of, or teased him or her in a hurtful way?” (c2) ‘‘excluded another student intentionally, or prevented another student from participating?” (d2) ‘‘made fun of with sexual jokes, comments or gestures to another students?” (e2) ‘‘blackmailed money from other students?” (f2) ‘‘bullied other students in some other way?”.

### Statistical analysis

The statistical analyses were conducted using SPSS 21.0 and SAS V.9.2. Descriptive analyses were used to describe the demographic characteristics and the prevalence of poor sleep quality and school bullying. The sleep quality differences between different groups were ascertained by a Chi-square test. Because our study used a multistage sampling method, the students were grouped into classes; therefore, they were not independent. Thus, multi-level logistic regression analyses were carried out to select the factors that may influence sleep quality. The GLMMIX procedure in SAS was used to fit the model in which classes were treated as clusters. A two-tailed *P*-value of less than 0.05 was considered significant for all tests.

## Results

### Descriptive characteristics of the participants by sleep quality

The descriptive characteristics of the participants by sleep quality are presented in Table 1. Among the 23,877 students, the mean age was 15.81±2.01 years; 46.27% of the students were boys, and 48.61% were junior high school students. A total of 6,127 students (25.66%) were reported to be poor sleepers. Among the participants, 11.65% and 40.06% had a poor economic status and high academic pressure, respectively. The proportion of participants who had poor relationships with their families, classmates, and teachers were 15.49%, 2.98%, and 5.40%, respectively. A total of 5.38% of the participants were smokers.

**Table 1:** Descriptive characteristics of the participants by sleep quality (n, %).

Variable	Total	Good sleep quality	Poor sleep quality	*Chi-Square*	*p*-value
Gender					
Boy	11049(46.27)	8340(46.99)	2709(44.21)	16.259	<0.001
Girl	12403(51.95)	9076(51.13)	3327(54.30)		
Grade					
Junior	11606(48.61)	9377(52.83)	2229(36.38)	500.388	<0.001
Senior	12061(50.51)	8212(46.26)	3849(62.82)		
Economic status					
Good	6509(27.26)	5174(29.15)	1335(21.79)		
General	14504(60.74)	10771(60.68)	3733(60.93)	285.335	<0.001
Bad	2781(11.65)	1746(9.84)	1035(16.89)		
Academic pressure					
Low	3520(11.74)	2880(16.23)	640(10.45)		
Middle	10767(45.09)	8629(48.61)	2138(34.89)	727.178	<0.001
High	9564(40.06)	6220(35.04)	3344(54.58)		
Relationship with families					
Good	18397(77.05)	14286(80.48)	4111(67.10)		
Average	4125(17.28)	2740(15.44)	1385(22.60)	562.180	<0.001
Bad	1312(5.49)	692(3.90)	620(10.12)		
Relationship with classmates					
Good	16552(69.32)	12872(72.52)	3680(60.06)		
Average	6533(27.36)	4401(24.79)	2132(34.80)	371.842	<0.001
Bad	711(2.98)	410(2.31)	301(4.91)		
Relationship with teachers					
Good	11897(49.83)	9531(53.70)	2366(38.62)		
Average	10619(44.47)	7442(41.93)	3177(51.85)	535.645	<0.001
Bad	1289(5.40)	722(4.07)	567(9.25)		
Smoking					
Yes	1284(5.38)	758(4.27)	526(8.58)	165.590	<0.001
No	22204(92.99)	16692(94.04)	5512(89.96)		
Bullying behaviors					
Victim	1410(5.91)	828(4.66)	582(9.50)		
Bully	401(1.68)	225(1.27)	176(2.87)	524.092	<0.001
Bully-victim	784(3.28)	402(2.26)	382(6.23)		
Neutral	21282(89.13)	16295(91.80)	4987(81.39)		

A higher proportion of girls were poor sleepers (51.30% vs. 54.30%, p<0.001), and a higher proportion of senior high school students were poor sleepers (46.26% vs. 62.82%, p<0.001). More poor sleepers suffered from poor economic statuses (9.84% vs. 16.89%, p<0.001) and high academic pressure (35.04% vs. 54.58%, p<0.001) and were involved in bullying (9.20% vs.18.61%, p<0.001); less poor sleepers currently had good relationship with their families (80.48% vs. 67.10%, p<0.001), classmates (72.52% vs. 60.06%, p<0.001) and teachers (53.70% vs. 38.62%, p<0.001).

Victimization and bullying were prevalent among high school students. Of the total participants, 10.89% reported being involved in school bullying during the past 30 days, with 1,410 (5.91%) of the students reporting being bullied and 401 (1.68%) admitting to bullying others. A subset of 784 (3.28%) students was involved in both victimization and bullying. As we can observe in Table 1, there were significant sleep quality differences among these different groups (p<0.001).

### Elements of sleep quality by involvement in bullying behaviors

Seven components of sleep quality in the present sample population are listed in Table 2. Of the total participants, 25.66% of the students reported having poor sleep quality, 20.93% of the students reported sleep latencies over 30 minutes, 25.42% of the students reported sleep durations less than 6 hours, 75.66% of the students reported sleep disturbances, 1.34% of the students had used sleep medication and 92.82% of the students reported having dysfunction during the daytime.

**Table 2:** Elements of sleep quality by involvement in bullying (n, %).

Elements of sleep quality	Total	Involvement in bullying	Neutral	Chi-Square	*p*-value
Subjective sleep quality					
very good	3282(13.75)	311(11.98)	2971(13.96)	481.162	<0.001
good	12415(52.00)	1009(38.88)	11406(53.59)		
poor	6878(28.81)	930(35.84)	5948(27.95)		
very poor	1302(5.45)	345(13.29)	957(4.50)		
Sleep latency					
≤15 min	9930(41.59)	824(31.75)	9106(42.79)	382.371	<0.001
16～30 min	8949(37.48)	888(34.22)	8061(37.88)		
31～60 min	3935(16.48)	621(23.93)	3314(15.57)		
≥60 min	1063(4.45)	262(10.10)	801(3.76)		
Sleep duration					
＞7 h	9304(38.97)	818(31.52)	8486(39.87)	202.142	<0.001
6～7 h	8504(35.62)	878(33.83)	7626(35.83)		
5～6 h	4781(20.02)	631(24.32)	4150(19.50)		
<5 h	1288(5.39)	268(10.33)	1020(4.79)		
Habitual sleep efficiency					
＞85%	21037(88.11)	2160(83.24)	18877(88.70)	95.189	<0.001
75%～85%	2027(8.49)	272(10.48)	1755(8.25)		
65%～75%	462(1.93)	85(3.28)	377(1.77)		
<65%	351(1.47)	78(3.01)	273(1.28)		
Sleep disturbance					
none	5567(23.32)	386(14.87)	5181(24.34)	759.281	<0.001
mild	16513(69.16)	1703(65.63)	14810(69.59)		
moderate	1656(6.94)	429(16.53)	1227(5.77)		
severe	141(0.59)	77(2.97)	64(0.30)		
Use of sleep medication					
none	23557(98.66)	2496(96.18)	21061(98.96)	253.808	<0.001
mild	184(0.77)	28(1.08)	156(0.73)		
moderate	50(0.21)	21(0.81)	29(0.14)		
severe	86(0.36)	50(1.93)	36(0.17)		
Daytime dysfunction					
none	1715(7.18)	141(5.43)	1574(7.40)	506.644	<0.001
mild	5915(24.77)	381(14.68)	5534(26.00)		
moderate	11348(47.53)	1125(43.35)	10223(48.04)		
severe	4899(20.52)	948(36.53)	3951(18.56)		

As these results indicate, the seven elements of sleep quality varied widely between those who were involved in bullying and those who were neutral. The students who were involved in bullying behaviors (being bullied or bullying others) reported a poorer subjective sleep quality, a longer sleep latency, a shorter sleep duration, less habitual sleep efficiency, more daytime dysfunction, an increased use of sleep medications and more sleep disturbances. The total measure of poor sleep quality occurred more frequently among the student who were bullied or bullies or both, and the differences were all significant.

### Frequency of bullying behaviors and poor sleep quality

The frequencies of different types of bullying behaviors (being bullied or bullying others) and total poor sleep quality are represented in Table 3. In our study, 670 (2.81%) of the students reported being physically bullied at least once or twice in the past 30 days; 5,701 (23.88%) reported being verbally bullied; and 1,809 (7.58%) reported being relationally bullied. A total of 634 (2.66%) of the students reported physically bullying others at least once or twice in the past 30 days; 3,144 (13.17%) reported verbally bullying; and 1,045 (4.38%) reported relationally bullying.

**Table 3:** The frequency of bullying behaviors by sleep quality (n, %).

Bullying behaviors	Total	Poor sleep quality	Good sleep quality	*Chi-Square*	*p-value*
Physical Victimization					
never	23207	5845(25.19)	17362(74.81)		
sometimes	411	145(35.28)	266(64.72)	123.382	<0.001
often	259	137(52.93)	122(47.17)		
Verbal Victimization					
never	18176	4131(22.73)	14045(77.27)		
sometimes	3067	899(29.31)	2168(70.69)	456.306	<0.001
often	2634	1097(41.65)	1537(58.35)		
Relational Victimization					
never	22068	5363(24.30)	16705(75.70)		
sometimes	1240	464(37.42)	776(62.58)	329.697	<0.001
often	569	300(52.72)	269(47.28)		
Physical Bullying					
never	23243	5866(25.24)	17377(74.76)		
sometimes	412	144(34.95)	268(65.05)	105.926	<0.001
often	222	117(52.70)	105(47.30)		
Verbal Bullying					
never	20733	4941(23.83)	15792(76.17)		
sometimes	1707	555(32.51)	1152(67.49)	329.285	<0.001
often	1437	631(43.91)	806(56.09)		
Relational Bullying					
never	22832	5671(24.84)	17161(75.16)		
sometimes	712	284(39.89)	428(60.11)	201.573	<0.001
often	333	172(51.65)	161(48.35)		

**Fig 1:**
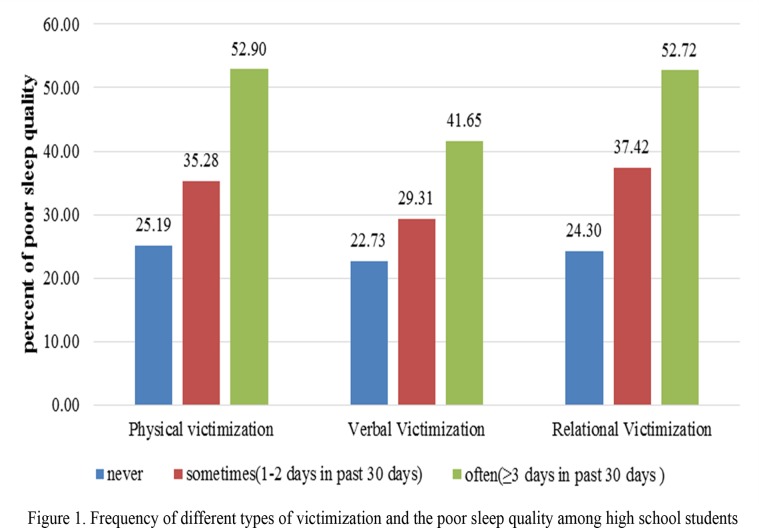
Frequency of different types of victimization and the poor sleep quality among high school students.

**Fig 2:**
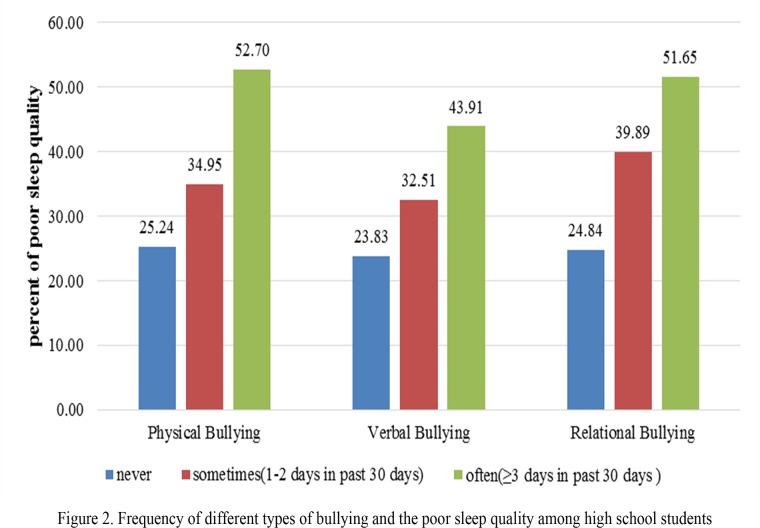
Frequency of different types of bullying and the poor sleep quality among high school students.

The students, who were involved in bullying behavior, whether as victim or a bully, were at a significantly higher risk for poor sleep quality compared with students who were never victims or bullies. For example, the incidence of poor sleep quality among the students who were never physically bullied was 25.19%, while among the students who were sometimes physically bullied, the incidence was 35.28%; the incidence among those who were often physically bullied was 52.90%. A similar pattern was found among verbal victimization, relational victimization and different forms of bullying others. The more frequent the involvement in bullying behavior (whether as a victim or a bully), the more likely the student had poor sleep quality (Figs. 1 and 2).

### Association of poor sleep quality and bullying behaviors

The final logistic regression model for poor sleep quality is presented in Table 4. After adjusting for gender, grade, economic status, academic pressure, relationships with families, classmates and teachers, and smoking, the students who were being bullied (OR = 2.05, 95% CI = 1.81–2.32), bullied others (OR = 2.30, 95% CI = 1.85–2.86) and both (OR = 2.58, 95% CI = 2.20–3.03) were at a higher risk for poor sleep quality. In addition, we conducted regression analysis to examine odds ratios associated with involvement in bullying behaviors predicting sleep duration, sleep latency, etc. Finally, our results indicated that bullying others, being bullied and victim-bully predicted all aspects of the sleep quality, including sleep duration, sleep latency, etc (Table 5).

**Table 4:** Association of the poor sleep quality and bullying behaviors.

Covariates		Reference	Adjusted OR	95% CI	*p*-value
Gender	Girl	Boy	1.32	1.23–1.41	<0.001
Grade	Junior	Senior	1.91	1.79–2.04	<0.001
Economic status	General	Good	1.04	0.97–1.14	0.211
	Bad		1.20	1.07–1.34	0.001
Academic pressure	Average	Low	1.18	1.06–1.32	0.002
	High		2.32	2.09–2.57	<0.001
Relationship with families	Average	Good	1.46	1.35–1.59	<0.001
	Bad		2.21	1.94–2.51	<0.001
Relationship with classmates	Average	Good	1.29	1.19–1.39	<0.001
	Bad		1.23	1.02–1.34	0.017
Relationship with teachers	Average	Good	1.31	1.22–1.41	<0.001
	Bad		2.00	1.73–2.30	<0.001
Smoking	Yes	No	1.67	1.47–1.91	<0.001
Bullying behavior	Victim	Neutral	2.05	1.81–2.32	<0.001
	Bully		2.30	1.85–2.86	<0.001
	Bully-Victim		2.58	2.20–3.03	<0.001

Notes: Adjusted for gender; grade; economic status; academic pressure; relationship with families, classmates, and teachers; and smoking

**Table 5:** The regression of seven aspects of the sleep quality.

	Covariates	Adjusted OR	95%CI	*p*-value
Subjective sleep quality	Victim	1.98	1.78–2.21	<0.001
	Bully	1.88	1.54–2.29	<0.001
	Victim-Bully	2.15	1.86–2.48	<0.001
Sleep latency	Victim	2.05	1.82–2.30	<0.001
	Bully	1.82	1.47–2.26	<0.001
	Victim-Bully	2.54	2.19–2.95	<0.001
Sleep duration	Victim	1.43	1.27–1.60	<0.001
	Bully	1.69	1.37–2.80	<0.001
	Victim-Bully	2.11	1.81–2.44	<0.001
Habitual sleep efficiency	Victim	1.76	1.37–2.25	<0.001
	Bully	2.75	1.90–3.98	<0.001
	Victim-Bully	2.49	1.88–3.30	<0.001
Sleep disturbances	Victim	3.47	3.00–4.01	<0.001
	Bully	3.05	2.33–4.00	<0.001
	Victim-Bully	4.68	3.93–5.58	<0.001
Use of sleep medication	Victim	2.57	1.81–3.65	<0.001
	Bully	2.94	1.63–5.30	<0.001
	Victim-Bully	6.49	4.74–8.90	<0.001
Daytime dysfunction	Victim	1.85	1.62–2.11	<0.001
	Bully	1.71	1.35–2.16	<0.001
	Victim-Bully	2.52	2.09–3.05	<0.001

Notes: Reference covariate: Neutral

## Discussion

The current cross-sectional study used the validated PSQI to evaluate the sleep quality in adolescent bullying profiles with a large sampled population in China. We found that 10.89% of the students were involved in bullying behavior and that the overall prevalence of poor sleep quality (defined as PSQI >7) was 25.66%. Previous studies have showed that the number of students involved in school bullying in different countries varies between 6.3 and 45.2% [15] and that the prevalence of sleep problems varies from 10 to 33% [28, 29]. Our results are in agreement with the results of these studies showing that bullying is a widespread phenomenon [25] and that poor sleep quality is highly prevalent in the adolescents [30].

After adjusting for age, sex, and other confounding factors, our finding showed that students who were being bullied (OR = 2.05, 95%CI = 1.81–2.32), bullied others (OR = 2.30, 95%CI = 1.85–2.86) or both (OR = 2.58, 95%CI = 2.20–3.03) were at a higher risk of poor sleep quality. In addition, our study found that more frequent exposure to bullying resulted in a higher risk of experiencing poor sleep quality. To date, few studies have examined the association between school bullying and sleep disturbances. The study by Kubiszewski *et al*. [31] showed that victims of bullying showed significantly more subjective sleep disturbances than the pure-bully or neutral groups in France, after controlling for the effects of gender and age. More recently, other studies have investigated the association between workplace bullying and sleep disturbances. Niedhammer *et al*. [22] found that the risk of poor sleep quality among those experiencing weekly (OR = 3.25, 95%CI = 2.27–4.66) and daily (OR = 6.34, 95%CI = 4.31–9.33) exposure to bullying was higher than those without exposure in the working population. Hansen *et al*. [32] found that those who were occasionally bullied had higher ORs of disturbed sleep (OR = 3.60, 95%CI = 1.31–9.86) and a poor quality of sleep (OR = 5.22, 95%CI = 1.69–16.0) compared with those who were not bullied. These studies all suggest that bullying is closely associated with poor sleep quality.

Our results showed that those who frequently bullied others reported a poorer sleep quality, which may be explained by the fact that sleep becomes disturbed by intrusive thoughts and ruminations before and after specific bullying episodes [33]. However, being a victim of bullying could also lead to poor sleep quality, which could be explained by alterations in the hypothalamic–pituitary–adrenal (HPA) axis that are produced by chronic stress related to bullying. The HPA axis is known to influence sleep [34].

A typical bully or victim is likely to experience difficulties in solving social problems and usually has negative attitudes and beliefs and poor interpersonal relationships [35]. Researchers have found an association between involvement in bullying and a number of social stresses and psychological symptoms, including depression, anxiety, fears of going to school, and feelings of being unsafe and unhappy at school [36–38]. In addition, students with chronic sleep problems usually report more negative emotional states such as feeling tired, tense, anxious, depressed, and inattentive and experiencing conduct disorder [39–42]. These social stresses show a strong association with sleep problems and greatly disturb sleep in adolescents, which may be a mechanism behind bullying and sleep difficulties [43]. Moreover, stressful situations may cause disturbed sleep and less refreshing sleep [44], which is an underlying mechanism that could involve a lack of self-confidence to address stressful situations and the use of ineffective coping strategies during peer confrontation [31]. In our society, the social stress experienced by victims of bullying could arouse powerful feelings of threat, and any perception of threat and the associated increased arousal will make it difficult to fall asleep and thereby decrease sleep quality [40]. Moreover, the alertness and stress response system increases significantly during adolescent development, which could lead adolescents to have a higher risk of bullying-related sleep impairments [31].

In the present study, there are some limitations that should be considered. First, the data that we collected were cross-sectional and can thus only be used to evaluate statistical relationships but not causation. Longitudinal research that further indicates the cause–result relation could be the focus of future studies. Second, the school bullying was self-reported, which may be subjectively biased and may over- or underestimate the associations between school bullying and sleep. Future studies should assess bullying behaviors using more objective measures. Finally, no details regarding specific time frame for demographic questions were in our questionnaire. We advocate that future investigation to give a specific time frame for demographic questions is recommended.

Despite these limitations, our results provided insight into the relationship between school bullying and sleep quality. While we cannot provide a determinate interpretation for this relationship, we can suggest several possibilities that could be benefited by future study. The findings are of potential practical importance both for parents who want to support their children’s socio-emotional development and well-being and for educational professionals who must deal with the students involved in bullying.

## Conclusions

In conclusion, we investigated the prevalence of school bullying and sleep quality among Chinese high school students using a large-scale, randomly selected sample and examined the association between these factors. The results showed that the sleep quality of high school students is not optimistic and that school bullying is prevalent among adolescents in China. Additionally, being involved in school bullying might be a risk factor for poor sleep quality in adolescents. Given the prevalence of bullying observed in this study and its potential damage on sleep quality, effective preventive intervention measures should require a full consideration of the social and environmental factors that would stop bullying behaviors among Chinese adolescents.
